# Quantitative proteomic analysis identifies the unfolded protein response as a host pathway co-opted by ASFV to promote replication

**DOI:** 10.1128/mbio.03242-25

**Published:** 2025-12-05

**Authors:** Danyang Zhang, Baohong Liu, Huanan Liu, Ruoqing Mao, Weijun Cao, Xiangle Zhang, Fayu Yang, Yichao Wang, Chaochao Shen, Shilei Zhang, Zixiang Zhu, Haixue Zheng

**Affiliations:** 1 State Key Laboratory for Animal Disease Control and Prevention, College of Veterinary Medicine, Lanzhou University, Lanzhou Veterinary Research Institute, Chinese Academy of Agricultural Sciences 111658 , Lanzhou, China; 2 African Swine Fever Regional Laboratory of China, Lanzhou Veterinary Research Institute, Chinese Academy of Agricultural Sciences 111658, Lanzhou, China; 3 Key Laboratory of Animal Virology of the Ministry of Agriculture, Lanzhou Veterinary Research Institute, Chinese Academy of Agricultural Sciences 111658, Lanzhou, China; 4 Gansu Province Research Center for Basic Disciplines of Pathogen Biology, Lanzhou, China; Tsinghua University, Beijing, China

**Keywords:** African swine fever virus, proteomics, unfolded protein response, replication

## Abstract

African swine fever (ASF), caused by the African swine fever virus (ASFV), is a highly contagious disease that affects pigs, resulting in substantial economic losses in the global pig industry. A comprehensive understanding of viral-host protein interactions can facilitate the discovery of therapies for viral infection. In this study, we employ a 4D label-free quantitative proteomics approach to profile a comprehensive protein dynamics analysis in ASFV-infected pigs, identifying over 6,000 proteins across multiple organs. Our results reveal coordinated interorgan responses characterized by inflammatory activation and interferon signaling in defense against ASFV. The protein-protein interaction network analysis uncovers ASFV-induced functional modules, including the unfolded protein response (UPR), innate immune signaling, and inflammation, which are conserved across tissues. Notably, ASFV robustly activates all three branches of the UPR both *in vivo* and *in vitro* to promote viral replication. Furthermore, we identify that the virus-encoded protein D117L interacts with multiple UPR-related host proteins, thereby directly triggering UPR activation. Collectively, this study delineates the organ-specific proteomic landscape of ASFV infection, providing valuable insights into virus-host interactions and offering potential therapeutic targets for ASF.

**IMPORTANCE:**

African swine fever virus (ASFV) has caused severe consequences for the global pig industry. In this study, we conducted a multi-organ proteomic analysis using a 4D label-free quantitative proteomics approach and mapped the organ-specific proteomic landscape during ASFV infection. This work overcomes the limitations of most existing studies, which are primarily restricted to *in vitro* cell models and provide a more comprehensive understanding of ASFV infection and pathogenesis. Notably, the viral D117L protein is identified as a critical modulator of host cellular responses, directly subverting the unfolded protein response (UPR) pathway through specific interactions with host UPR-associated proteins. Collectively, our work lays the foundation for understanding the pathogenesis of ASFV, providing potential therapeutic strategies against African swine fever.

## INTRODUCTION

African swine fever (ASF) is a contagious viral disease of domestic and wild pigs caused by the African swine fever virus (ASFV), a large nucleocytoplasmic double-stranded DNA virus with a genome of approximately 170–193 kb in length, a multi-layered structure, and more than 150 proteins (1). In acute infection of highly virulent strains, splenomegaly is one of the most noticeable gross lesions with an enlarged, dark-colored, and friable spleen. Hemorrhagic lymphadenitis is commonly observed, particularly in the renal and lymph nodes (2, 3). ASFV causes extensive lymphoid depletion and apoptosis, especially in the spleen and lymph nodes (3, 4). It has high mortality, causing substantial economic losses to the swine industry worldwide (5–7). To date, no safe and effective vaccines or drugs are available to prevent or treat ASF due to the complexity of the virus entry, replication, assembly, and release, as well as its manipulation of host cell signaling and regulation of host cellular responses (8).

As the primary executors of biological function, proteins play very important roles in organisms. Proteomics is a powerful tool for large-scale protein identification and has been widely used in infectious disease research, drug development, and vaccine discovery. It has been adopted to assess the roles of proteins under numerous conditions, such as the different infection statuses, cell lines, viral strains, and host-pathogen interactions (9–13). As early as 2001, Rodriguez et al. used high-resolution two-dimensional electrophoresis to study the protein synthesis in porcine alveolar macrophages (PAMs) and Vero cells infected with ASFV (14). With the rapid development of molecular biology techniques, more and more high-throughput proteomics approaches have been developed. Tandem mass tag-based quantitative proteomics has been applied to ASFV-infected PAMs and tissues (15, 16), while iTRAQ combined with liquid chromatography-tandem mass spectrometry (LC-MS/MS) has identified proteins in bone marrow-derived macrophages (BMDMs) (17) and serum from ASFV-infected pigs (18). Stable isotope labeling by amino acids in cell culture is based on the metabolic incorporation of heavy isotope-labeled amino acids into the newly synthesized protein during cell culture. It was used in ASFV-infected macrophages and revealed that the host protein synthesis was broadly suppressed, with considerable variability across different proteins (19). However, proteomic studies analyzing the host protein expression after ASFV infection on a global scale, especially *in vivo*, are very limited (19). The 4D label-free quantitative proteomics is a revolutionary technology that incorporates a fourth dimension of ion mobility into the retention time, mass-to-charge ratio, and ion intensity dimensions (20, 21). This additional dimension enables precise differentiation of structurally similar peptides based on their ‌collision cross-sectional area. The high sensitivity and accuracy of the 4D label-free technique enable the detection of low-abundance but potentially functional proteins that are difficult to identify using conventional 3D proteomics, making it a promising tool for analyzing complex biological samples (20–22). It has become a high-throughput screening technology widely used in quantitative proteomics.

In this study, we employed 4D label-free LC-MS/MS to profile the proteome in ASFV-infected tissues of pigs. Bioinformatics analysis based on the proteomics results was used to identify proteins involved in the pathogenic mechanism of ASFV-associated lesions in different tissues. The results showed that different tissues cooperated in responses to ASFV infection and coordinated the defense against ASFV through inflammatory responses and interferon activation. The unfolded protein response (UPR) was also found to be activated across all three tissues. UPR is a conserved response in eukaryotes that alleviates endoplasmic reticulum (ER) stresses induced by various abiotic and biotic factors (23). Many viruses are capable of eliciting the UPR in host cells, and this response has been found to play critical roles in viral infection (24–26). Three signaling pathways, comprising eukaryotic translation initiation factor 2 alpha kinase 3 (EIF2AK3/PERK), ER to nucleus signaling 1 (ERN1/IRE1), and activating transcription factor 6 (ATF6), are essential for the UPR (27). Galindo et al. revealed that the ATF6 branch of the UPR and apoptosis were activated in Vero cells to promote ASFV infection (28). Zhong et al. found that ASFV MGF110-7L induced host cell translation suppression and stress granule formation by activating the PERK/PKR-eIF2a pathway *in vitro* (29). In the present study, UPR was exploited by ASFV to facilitate its replication. These findings advance our understanding of ASFV-host interactions at the systems level and may provide potential targets for the early diagnosis and treatment of ASF, ultimately contributing to the development of anti-ASFV drugs.

## RESULTS

### Proteomic profiles of pig kidney, submandibular lymph node, and spleen after ASFV infection

**Fig 1:**
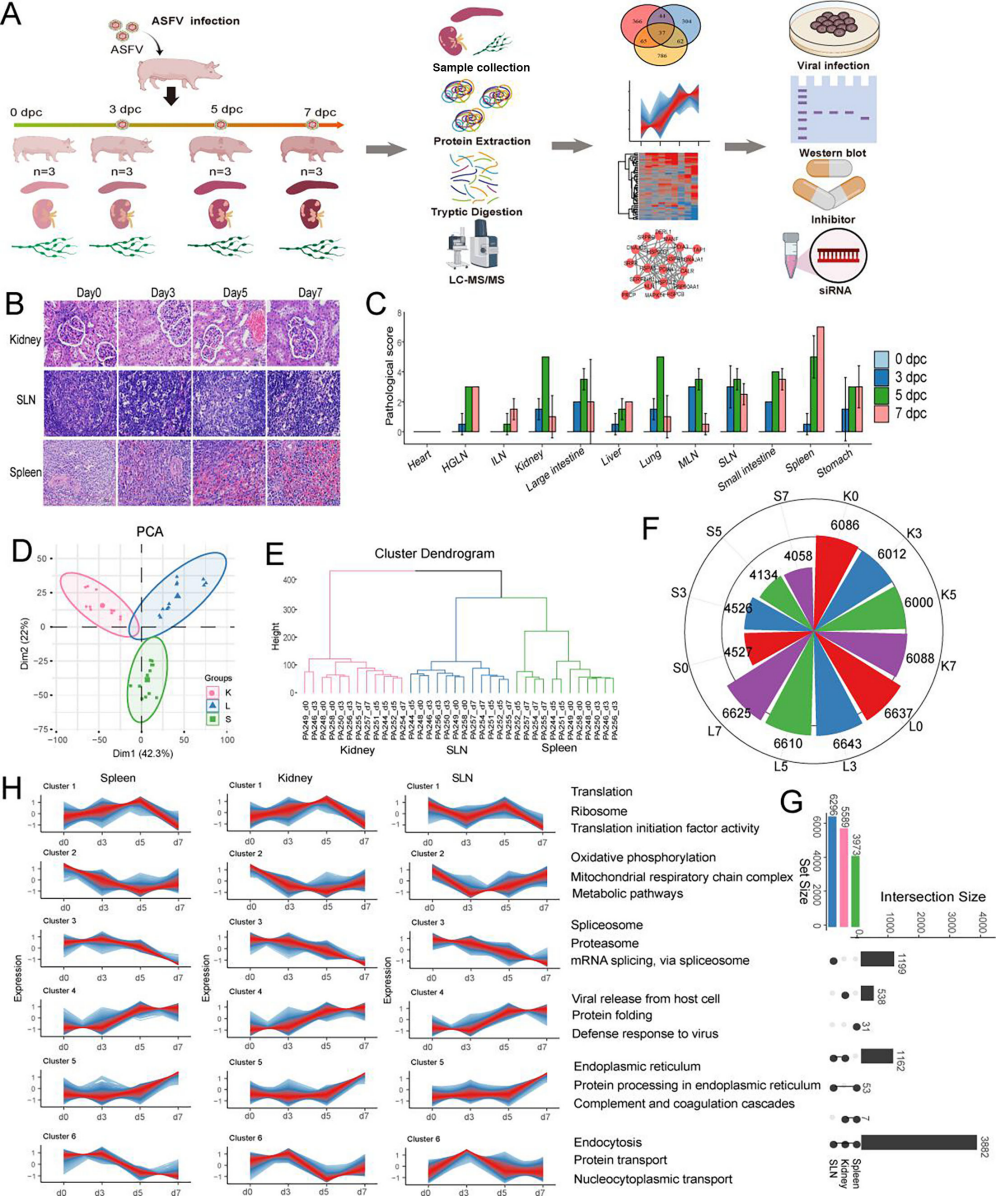
Proteomic profiles of pig kidney, SLN, and spleen after ASFV infection. (**A**) The workflow schematic of this study. The tissue samples were collected at the indicated time courses after ASFV challenge to identify the critical host pathways or proteins involved in virus infection by quantitative proteomic analysis. (**B and C**) The pathological features and scores of various organs, including the kidney, SLN, and spleen, were examined throughout the ASFV infection. (**D**) Principal component analysis showed a high correlation between biological replicates and clear separations among tissues. (**E**) Hierarchical clustering for all 36 samples. (**F**) Quantified proteins for each time point in each tissue. (**G**) The intersection proteins across three tissues by UpSet plot. (**H**) The time series profile clusters identified by TCseq. HGLN, hepatogastric lymph node; ILN, inguinal lymph node; K, kidney; L, SLN; MLN, mesenteric lymph node; S, spleen.

To profile and systematically characterize the organ-level changes of protein expression following ASFV infection, we examined both the tissue distribution of the virus and the associated histopathological changes (Fig. 1A). Significant pathological changes occurred in the major organs of infected pigs, characterized by extensive necrosis, cellular degeneration, and infiltration of inflammatory cells (Fig. 1B). As the infection progressed, the kidneys exhibited prominent tubular epithelial cell necrosis and fragmentation, accompanied by marked congestion in the glomerular capillaries and interstitial arterioles. Severe lymphocyte necrosis and disintegration were observed in the submandibular lymph nodes (SLNs). The spleen was also extensively damaged, with pronounced hemorrhage in both the red and white pulps, as well as widespread necrosis of lymphocytes and macrophages (Fig. 1B).

Given that significant pathological lesions were observed in the kidney (K), SLN (L), and spleen (S) (Fig. 1C), we used a 4D label-free MS-MS strategy to quantify the proteome of these organs following ASFV challenge at 0, 3, 5, and 7 days post-infection, with three biological replicates per time point. Principal component analysis and hierarchical clustering showed high reproducibility among replicates and clear separations between different tissues (Fig. 1D and E). We analyzed the proteins identified at each time point in each tissue to assess the core proteome throughout ASFV infection. The SLN exhibited the highest number of consistently detected proteins across all time points (6,296), followed by the kidney (5,589) and the spleen (3,973). In total, 3,882 proteins were discovered to be widely expressed across all time points and all three tissues (Fig. 1F and G).

To better dissect genome-wide protein expression dynamics during ASFV infection, we applied TCseq to analyze the 3,882 common proteins and identified six distinct expression clusters, each representing a group of proteins with similar temporal expression patterns across the four time points in each tissue (Fig. 1H). In cluster 1, protein levels increased slightly before declining at 7 dpi, and these proteins were mainly involved in translation, ribosomal structure, and initiation factor activity. Proteins in cluster 2 were associated with oxidative phosphorylation, which showed reduced expression following ASFV infection. Cluster 3 included proteins engaged in the spliceosome, mRNA splicing, and proteasome, whose expression gradually decreased over time. Cluster 4 consisted of proteins with increasing expression involved in viral release from the host cell, protein folding, and defense response to the virus. Proteins in cluster 5 remained stable until a marked increase at 7 dpi, primarily participating in protein processing in the endoplasmic reticulum. Cluster 6 contained proteins with functions of endocytosis, protein transport, and nucleocytoplasmic transport and showed a high expression at 3 dpi.

### ASFV infection drives widespread dysregulation of cellular pathways and functions

**Fig 2:**
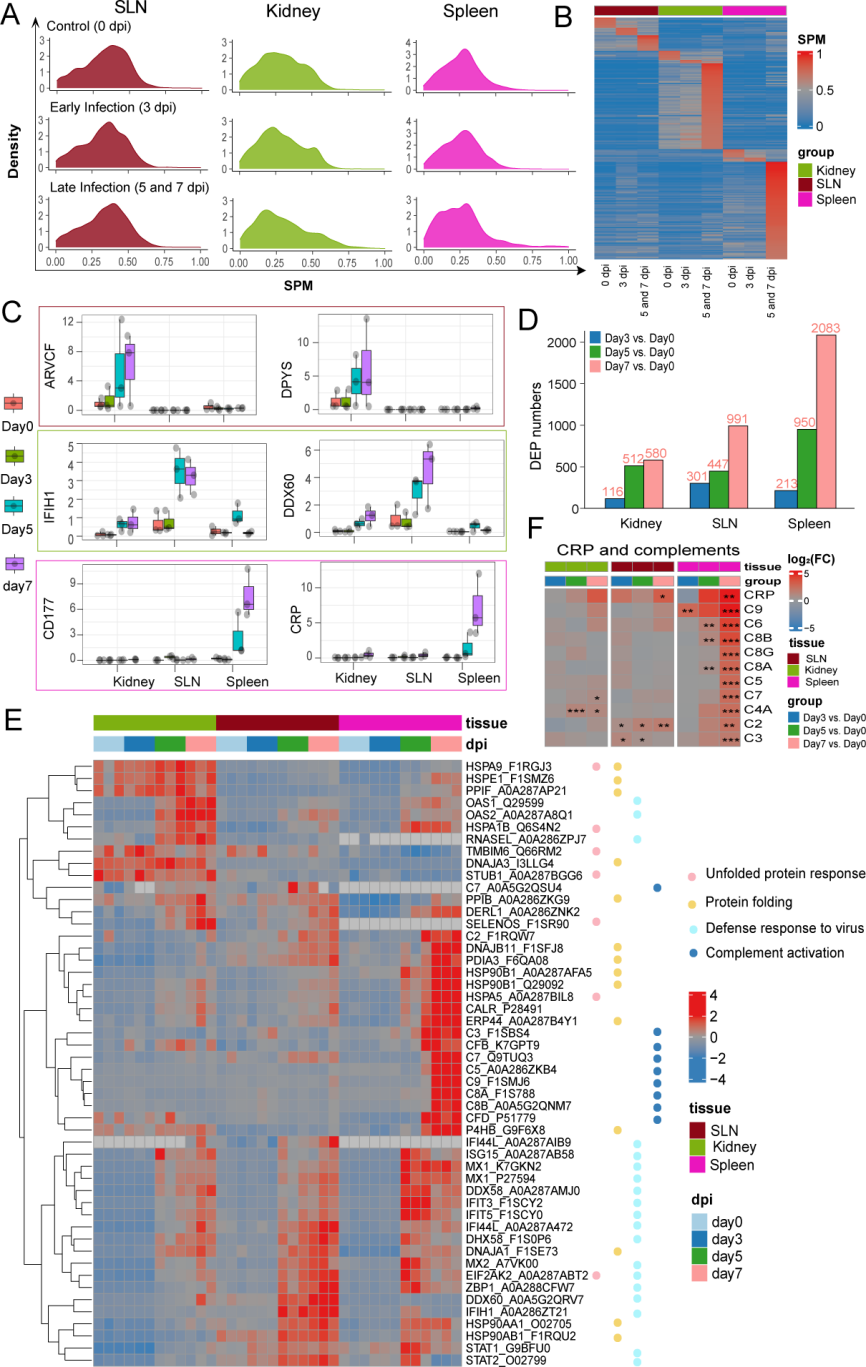
Tissue-specific infection-related protein identification. (**A**) Density plot showing the distribution of organ specificity (SPM) in the three infection stages of all proteins identified in the proteome. (**B**) Heatmap of protein SPM scores over 0.7 in each scenario. (**C**) Boxplots of the top two late infection stage-specific proteins for each tissue. (**D**) The barplot of differentially expressed proteins (DEPs) in each tissue. (**E**) Heatmap of log2 fold changes (FCs) of the complements compared to day 0 in each tissue. (**F**) Heatmap of 50 selected proteins whose regulation concentrated on four enriched pathways.

Tissue-specific proteins at each infection stage were identified by the specificity measure (SPM) method. As shown in Fig. 2A, the distribution of SPM scores varied across infection stages, with a cutoff of 0.7 applied to define tissue-specific proteins. SLN exhibited the fewest specific proteins regardless of infection stages, whereas the kidney and spleen showed a significantly higher number of specific proteins in the late phase (Fig. 2B). Specifically, ARVCF and DPYS proteins were predominantly expressed in the kidney in the late infection stage. The expression level of IFIH1 and DDX60 in the late infection stage of the SLN exceeded all other stages and tissues. In the spleen, the highest expression levels were observed for CD177 and C-reactive protein (CRP) (Fig. 2C).

Differential protein expression analysis showed that the spleen contained the largest number of differentially expressed proteins (DEPs), especially at 7 dpi (Fig. 2D). Functional enrichment revealed these DEPs were highly involved in ER protein processing, translation, protein folding, innate immunological response, and antiviral response. Among them, unfolded protein response, protein folding, defense response to virus, and complement activation were significantly dysregulated (Fig. 2E). CRP, identified as a spleen-specific marker during late-stage infection, was markedly upregulated at 7 dpi (Fig. 2C). Given that CRP activates the complement system to protect against infections (30), we further examined the complement system and found that all the complement components were upregulated in the spleen after infection on day 7 (Fig. 2F).

**Fig 3:**
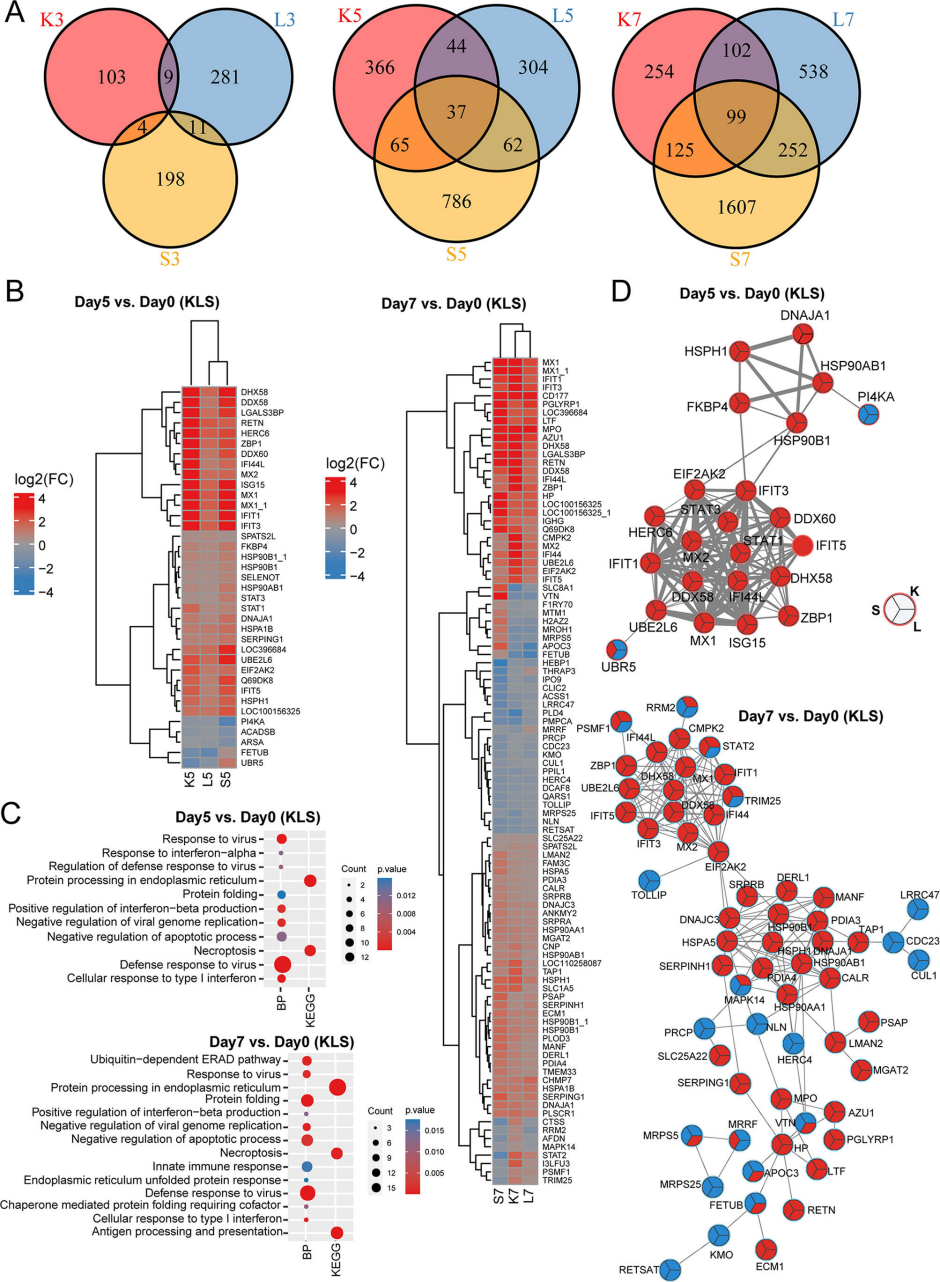
Dysregulated functional modules for DEPs. (**A**) The Venn diagram of DEPs in each infection stage. (**B**) The heatmap of DEPs for common DEPs across tissues. (**C**) The functional enrichment analysis results for common DEPs. (**D**) Functional modules constructed for common DEPs by PPI networks at 5 and 7 dpi. Different partitions of the circle represent different tissues. Red represents upregulation, and blue represents downregulation.

No intersected DEPs were detected across the three tissues at 3 dpi, whereas a total of 37 and 99 common DEPs were identified at 5 and 7 dpi, respectively (Fig. 3A). These common DEPs were mostly upregulated upon infection and functionally involved in innate immune response and protein folding pathways (Fig. 3B and C). Protein-protein interaction (PPI) networks of the intersected DEPs at 5 and 7 dpi were constructed using the STRING database to predict their functional interactions. Two protein modules were identified in each PPI network: one involved in protein folding and the other engaged in innate immune response (Fig. 3D). Interestingly, both modules were connected by EIF2AK2, a central kinase in cellular response to different stress signals that was significantly upregulated at both 5 and 7 dpi.

### ASFV activates ER stress and unfolded protein response *in vivo* and *in vitro*

**Fig 4:**
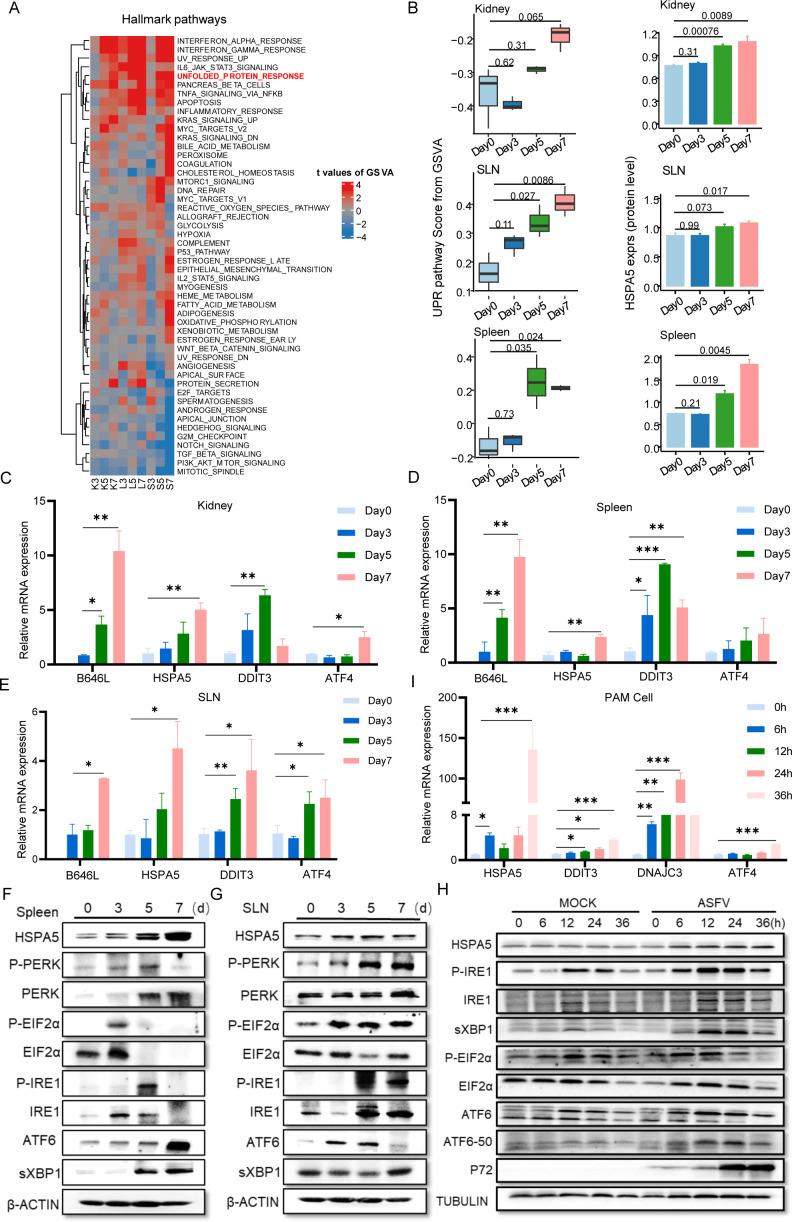
ASFV activates ER stress and unfolded protein response (UPR) *in vivo* and *in vitro.* (**A**) Heatmap of GSVA *t* values for hallmark pathways between each infection stage and baseline (day 0). (**B**) The boxplots of GSVA scores for the UPR pathway and the expression levels of HSPA5 across infection in each tissue. (**C–E**) mRNA expression analysis of ASFV gene B646L and the genes related to the UPR pathway (Hspa5, Ddit3, and Atf4) in the kidney, spleen, and SLN. (**F and G**) UPR signaling pathway activation in the spleen and SLN upon ASFV infection was examined by immunoblotting assay. (**H**) mRNA expression analysis of Hspa5, Ddit3, DNAJC3, and Atf4 in pig pulmonary alveolar macrophage (PAM) cells. (**I**) Immunoblotting analysis of UPR signaling pathway activation in ASFV-infected PAM cells. Data are presented as mean + SD. *P* values were calculated by a two-tailed unpaired *t*-test. *P  <  0.05, **P  <  0.01, ***P  <  0.001.

To characterize the biological pathways activated following ASFV infection, we performed gene set variation analyses (GSVAs) and found that the UPR was robustly activated upon infection, as well as the innate immune response, such as interferon response (Fig. 4A). As a major ER chaperone protein, HSPA5 (GRP78) has been generally accepted as a canonical marker of ER stress-activated UPR that maintains ER homeostasis (31). In line with the UPR scores, HSPA5 expression was significantly upregulated at 5 and 7 dpi across the three tissues, indicating that ASFV elicits ER stress *in vivo* (Fig. 4B). To validate this, HSPA5 protein and mRNA levels were determined by qPCR and Western blot. They were found to be significantly increased upon infection across all three tissues (Fig. 4C through G). Given that UPR includes three molecular branches (IRE1, PERK, and ATF6) (32), we further examined whether UPR activation by ASFV is branch specific. The results revealed that ASFV infection induced PERK-eIF2 and IRE1 phosphorylation, enhanced the splicing and expression of sXBP1 downstream of IRE1, and yielded more cleaved ATF6, indicating that all three branches of the UPR are activated (Fig. 4F and G; Fig. S1A).

To further confirm that ASFV infection activated ER stress, the transcriptional and translational levels of HSPA5 were also monitored in the ASFV-infected PAM cells. Consistent with the *in vivo* results, the HSPA5 protein level increased significantly along with the infection in PAM cells (Fig. 4H). We next examined UPR activation in PAM cells after infection with ASFV. Immunoblotting results showed that the phosphorylation of PERK, spliced XBP1 expression, and cleavage of ATF6 were increased in ASFV-infected PAMs, indicating that all three UPR branches were activated (Fig. 4H). Consistently, genes downstream of UPR, particularly the PERK-eIF2 branch, including DDIT3, ATF4, and DNAJC3, were also induced upon ASFV infection in tissues (Fig. 4C through E) and cells (Fig. 4I). Collectively, ASFV infection induced ER stress and triggered the UPR *in vitro* and *in vivo*.

### D117L activates three UPR pathways through targeting key UPR components

**Fig 5:**
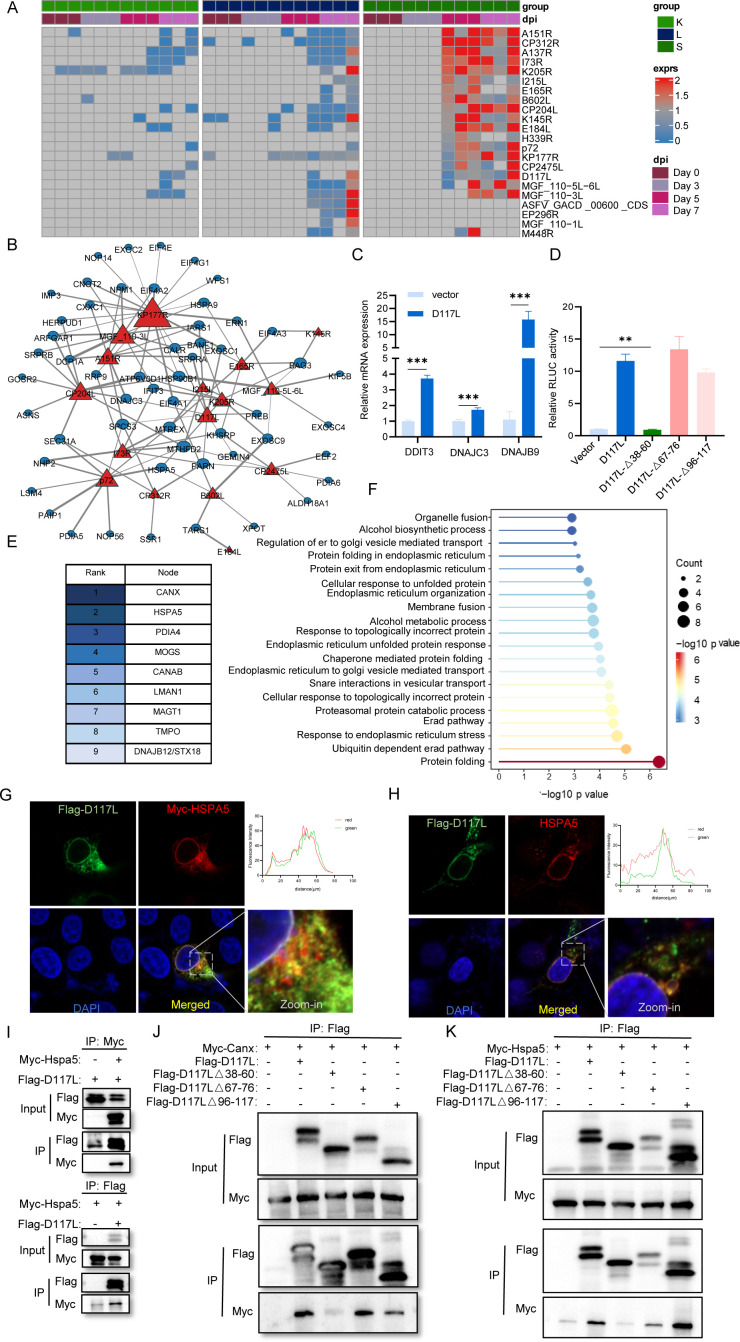
D117L activates three UPR pathways through targeting key UPR components. (**A**) Heatmap of ASFV proteins identified by proteomics analysis across infection. (**B**) PPI network analysis of the virus-host interaction. (**C**) HEK293T cells were transfected with vector or Flag-D117L for 24 h, and mRNA expression of Ddit3, Dnajc3, and Dnajb9 was detected by RT-qPCR. (**D**) HEK293T cells were transfected with vector, Flag-D117L, Flag-D117LΔ38-60, Flag-D117LΔ67-76, or Flag-D117LΔ96-117; each group was also co-transfected with ATF6-LUC and Renilla-TK. After 24 h post-transfection, luciferase activities were examined. (**E**) Mass spectrometry was performed to identify the interacting proteins of D117L and D117LΔ38-60, and the top 10 proteins were listed. (**F**) Kyoto Encyclopedia of Gene and Genomes pathway enrichment analysis of D117L interacting proteins. (**G and H**) Immunofluorescence analysis of the colocalization of Flag-tagged D117L (green) and Myc-tagged or endogenous HSPA5 (red) in iPAM cells. (**I**) Immunoprecipitation (IP) assay examining the interaction between HSPA5 and D117L. (**J and K**) IP assays assessing the interaction of D117L (WT) or D117LΔ38-60, D117LΔ67-76, and D117LΔ96-117 mutants with CANX or HSPA5 in HEK293T cells.Data are presented as mean + SD. PP values were calculated by a two-tailed unpaired t-test. **P  <  0.01, ***P  <  0.001.

ASFV has a large double-stranded DNA genome encoding more than 150 proteins (1). In our proteomics analysis, 22 ASFV proteins were identified, including both structural proteins and nonstructural proteins (Fig. 5A). The co-expression network between these viral proteins and the UPR-related proteins was constructed to explore their interactions by Pearson correlation coefficients (PCCs). Significant interactions were defined by a PCC >0.49 and a threshold of *P* value <0.05 (Fig. 5B). In the co-expression network, the triangles represent virus proteins, and the circles represent the host proteins. The degrees for the virus proteins were defined by the number of connections with the host proteins in the network. Then, the top 10 virus proteins were filtered out based on network degrees (Fig. S1B). In order to determine the effects of the above viral proteins on UPR, we simultaneously introduced these viral proteins into 293T cells and evaluated the activation of UPR. Interestingly, viral proteins CP204L, B646L, D117L, and A151R robustly increased the expression level of UPR marker HSPA5, indicating that UPR was significantly activated (Fig. S1C). To further validate this, an ATF6 luciferase reporter system was used. Dual-luciferase assays showed that D117L exhibited the strongest activation effect on the ATF6 gene (Fig. S1D). Once activated, the UPR promotes many downstream genes to maintain endoplasmic reticulum homeostasis. We further examined the transcriptional changes of the UPR downstream genes DDIT3, DNAJC3, and DNAJB9 and found that the D117L protein significantly enhanced their transcription (Fig. 5C). These results indicated that D117L is the predominant factor driving ASFV-induced UPR.

D117L has a transmembrane domain (A39-Y59) that is required for its interaction with STING (33). The UPR is initiated by the transmembrane proteins within the ER, like GRP78, PERK, IRE1, and ATF6. Since D117L can activate all branches of UPR, we hypothesize that the transmembrane domain of D117L is required for the activation of the UPR. We constructed a series of D117L deletion mutants and examined whether any domain is involved in UPR activation (Fig. S2A). The qPCR analysis revealed that DNAJC3 expression could not be induced in cells transfected with the D117L mutant lacking amino acids 38–60 (Fig. S2B). Consistently, the ATF6 luciferase assay further supported the critical role of 38–60 amino acids in mediating the activation of the UPR (Fig. 5D). To investigate the mechanism by which D117L activates UPR, we first analyzed the different physical binding proteins of the D117L and D117L mutants (38–60 aa deletion) through mass spectrometry. Notably, several UPR-related proteins were found as partners of D117L, including HSPA5, CANX, and PDIA4, but not for the D117L mutant (Fig. 5E and F). Among them, HSPA5 is a master regulator of the UPR (34), while CANX is a ubiquitously expressed molecular lectin-like chaperone that plays an important role in the ER-associated degradation pathway and the UPR (35). To validate these protein-protein interactions, we performed a reciprocal immunoprecipitation analysis and further confirmed the independent interactions between D117L and HSPA5 (Fig. 5I), as well as between D117L and CANX (Fig. S2C). However, no interaction between D117L and PDIA4 was observed (Fig. S2D). Consistently, the immunofluorescence assay results also showed that the D117L protein was colocalized with exogenous and endogenous HSPA5 (Fig. 5G and H). Next, the indicated deletion mutants of D117L were used for the immunoprecipitation analysis to map the interaction domains between D117L and its partners. Interestingly, we observed that the D117L mutant lacking amino acids 38–60 failed to pull down CANX and HSPA5, while mutants with deletions of amino acids 67–76 or 97–117 efficiently immunoprecipitated CANX and HSPA5 (Fig. 5J and K). These results indicate that D117L is crucial for ASFV-induced activation of UPR.

### Activation of UPR facilitates ASFV replication

**Fig 6:**
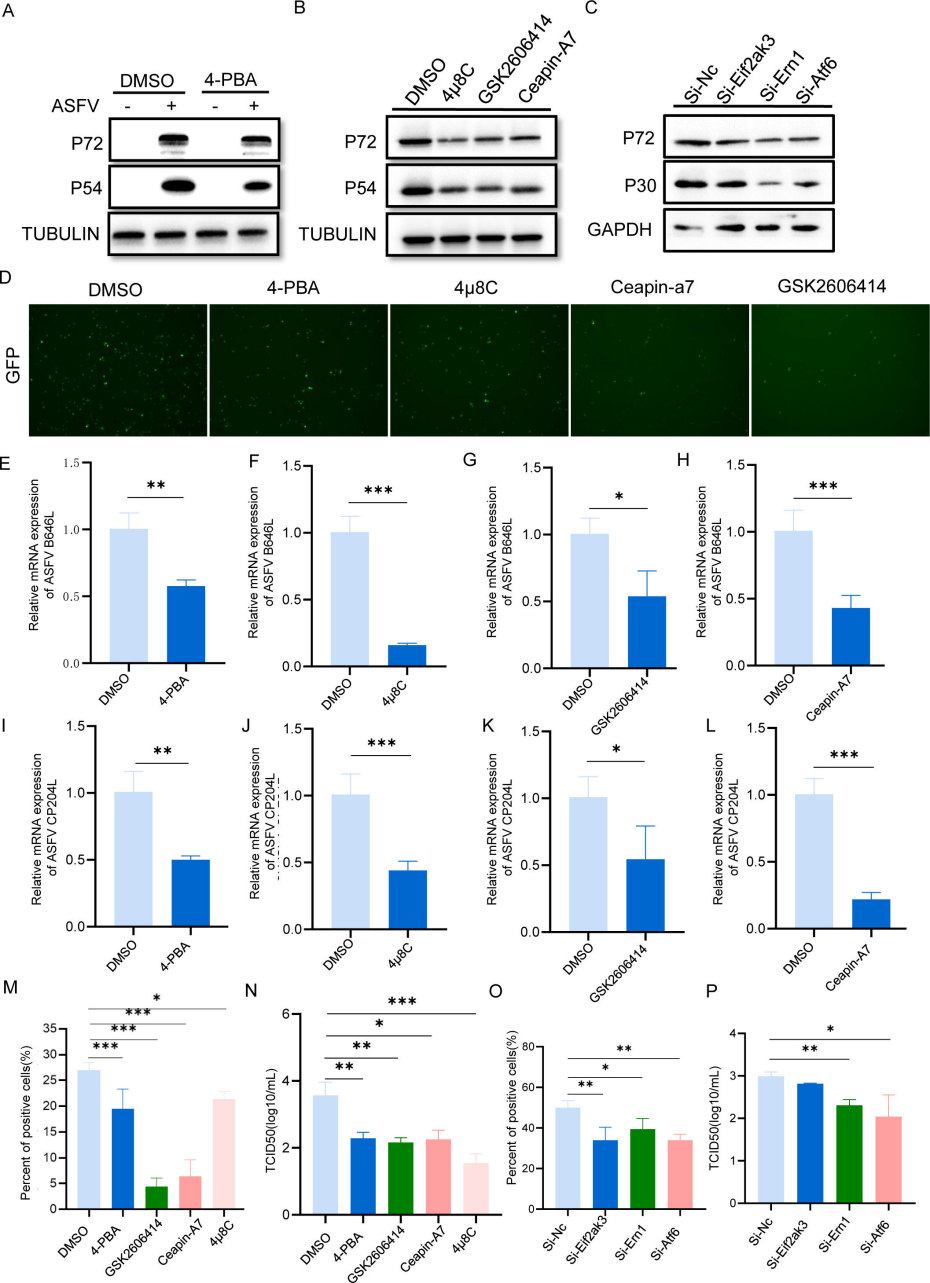
Activation of UPR facilitates ASFV replication. (**A**) PAM cells were treated with or without inhibitor 4-PBA (50 µM), followed by ASFV challenge at 0.1 MOI for 24 h; P72 and P54 proteins were detected by Western blotting; and tubulin was used as a loading control. (**B**) PAM cells were treated with or without inhibitors, 4μ8C (5 µM), GSK2606414 (0.5 µM), or Ceapin-A7 (5 µM), and then infected with ASFV at 0.1 MOI for 24 h. P72 and P54 proteins were detected by Western blotting. (**C**) PAM cells were transfected with siRNA-NC (negative control), siRNA-Eif2AK3, siRNA-ERN1, or siRNA-ATF6 for 24 h and then challenged with ASFV at 0.1 MOI. P72 and P30 proteins were detected by Western blotting at 24 h; GAPDH was used as a loading control. (**D**) Fluorescence analysis of ASFV replication in PAM cells pretreated with or without inhibitors, 4μ8C (5 µM), GSK2606414 (0.5 µM), or Ceapin-A7 (5 µM). (**E–L**) The antiviral activities of the inhibitors against ASFV mRNA transcription. Relative mRNA expression levels of ASFV genes B646L and CP204L in PAM cells were quantified. PAM cells were treated with or without inhibitors or transfected with siRNAs and then infected with GFP-ASFV at 0.1 MOI for 24 h. Flow cytometry was used to analyze the percentage of infected positive cells (**M and O**). Viral titers were measured by TCID_50_ assay at 72 h (**N and P**). Data presented as means + SD. *P* values were calculated by a two-tailed unpaired *t*-test. *P  <  0.05, **P  <  0.01, ***P  <  0.001.

Given the pivotal role of the ER in virus-host interactions (36), we investigated whether ER stress-activated UPR contributed to ASFV replication. As shown in Fig. 6A, treatment with the ER stress inhibitor 4-phenylbutyric acid (4-PBA) (37) effectively suppressed viral protein p72 and p54 synthesis, indicating the inhibitory activity of 4-PBA against ASFV. Next, we determined which UPR branches were required for ASFV replication. Three reagents (GSK2606414 [38], 4μ8C [39], and Ceapin-A7 [40]) were used to block PERK, IRE1, and ATF6 signaling activation. PAM cells were treated with the inhibitors, followed by infection with ASFV. We observed that all three inhibitors can suppress viral protein expression (Fig. 6B). Furthermore, the antiviral activities of these branch inhibitors were not attributable to cytotoxicity, as no significant cytotoxicity was observed by measuring cell viability with the CCK-8 assay (Fig. S3A through D). Similar results were obtained from the fluorescence assay (Fig. 6D). To further confirm this, siRNAs (si-Eif2AK3, si-ERN1, and si-ATF6) targeting each UPR signal sensor were also tested in PAM cells. The expression of the EIF2AK3 gene was reduced by only about 20%, whereas the interference efficiencies of the other two genes exceeded 70% (Fig. S3E through G). The results showed that si-ERN1 and si-ATF6 significantly inhibited the production of viral p30 and p72 (Fig. 6C). The effect of si-EIF2AK3 on viral replication was relatively weaker than that of the other two groups due to its limited interfering efficacy. In addition, the transcription of viral genes was also examined and yielded similar results, providing evidence for the anti-ASFV activities of UPR inhibitors (Fig. 6E through L). The antiviral activities of the inhibitors and the siRNAs were also evaluated by the flow cytometry assay, which revealed that the infection efficiency of GFP-ASFV was reduced when treated with inhibitors (Fig. 6M) or siRNAs (Fig. 6O). Furthermore, inhibition of either ER stress or individual UPR branches resulted in a decrease in the production of infectious virions determined by the TCID_50_ assay (Fig. 6N and P). Taken together, these findings demonstrate that ASFV-activated UPR is critical for efficient viral replication.

## DISCUSSION

The host’s translational machinery can be hijacked by the viruses to facilitate their replication (41). At the same time, the host cells develop various antiviral mechanisms upon sensing the viral genetic material (42, 43). An imbalance between the viral infection and host regulation may lead to cell death or virus clearance. Therefore, exploring the host-virus interaction is critical for understanding the pathogenic mechanisms of the virus (44). In this study, to investigate how the host responds to ASFV infection, we used 4D label-free LC-MS/MS quantification to compare the protein expression profiles of ASFV-infected samples across different infection stages and tissues. TCseq analysis of 3,882 common proteins revealed similar expression patterns across tissues, suggesting synergistic responses of different tissues against ASFV (16). Despite this coordinated response, there were still some considerable tissue and infection-specific proteins. As the mediator of the acute-phase response, CRP is known as a biomarker of injury, infection, and inflammation (45). In the present study, CRP was identified as a spleen-specific marker during the late phase of ASFV infection, aligning with the clinical sign that the spleen had the most severe lesions. CRP can activate the classical complement pathway (46), which is critical for innate immune responses but can cause detrimental tissue injury if dysregulated. The complement proteins were significantly upregulated in the spleen upon ASFV infection, especially at 7 dpi, when the highest levels of viral DNA were also detected, indicating that the CRP and complement system may play important roles in ASFV pathogenesis within the spleen.

The PPI network of differentially expressed proteins revealed two modules associated with innate immune response and protein folding, both of which were linked to EIF2AK2. EIF2AK2 is a serine/threonine protein kinase that is activated by double-stranded RNA (dsRNA), exerting antiviral activity against a wide range of DNA and RNA viruses, including West Nile virus, Sindbis virus, foot-and-mouth disease virus, Semliki Forest virus, and lymphocytic choriomeningitis virus. Upon activation, EIF2AK2 phosphorylates the translation initiation factor eIF2α to shut down host translational machinery, thereby limiting virus protein synthesis. Although the ASFV genome is double-stranded DNA, we reported that ASFV AT-rich islands can be transformed from dsDNA to dsRNA by RNA Pol-III, which in turn activates RIG-I-mediated antiviral innate response. Whether and how EIF2AK2 is activated by ASFV and whether dsRNA produced by RNA Pol-III also serves as a ligand for EIF2AK2 need to be further explored.

In this study, we demonstrated that the UPR signaling pathway was activated by ASFV infection both *in vivo* and *in vitro*. At least 10 ASFV proteins were identified as being associated with the UPR signaling pathway. Among them, K205R has been demonstrated to trigger ER stress and activate the unfolded protein response by activating the three branches of the UPR pathway (47). Consistent with the report by Xia et al. that D117L inhibits cell proliferation through ER stress-ROS-mediated cell cycle arrest (48), we also identified D117L, through a function-based screen, as a predominant ASFV protein activating the UPR. Mechanistically, D117L interacts with multiple UPR-related proteins, particularly HSPA5 (also known as BiP/GRP78), a key molecular chaperone involved in maintaining ER homeostasis. This interaction suggests that D117L may hijack host cell stress response machinery to benefit ASFV replication. While these findings provide novel insights into ASFV-host interactions, further studies are needed to delineate the precise molecular mechanisms by which D117L modulates UPR signaling.

Collectively, we revealed the global landscape of proteins, regulatory modules, and pathways that are dysregulated by ASFV infection. Network-based analyses combined with experimental validation identified ER stress-activated UPR as a key host response opted by ASFV to facilitate replication. These findings broadened our understanding of ASFV infection and provide potential therapeutic targets against ASFV.

## MATERIALS AND METHODS

### Animals, cells, and viruses

Thirty-three pigs (age, 10 weeks; weight, ~40 kg) were used in the present study. All the used animals were free from ASFV, classical swine fever virus, porcine epidemic diarrhea virus, porcine reproductive and respiratory syndrome virus, pseudorabies virus, porcine parvovirus, porcine circovirus 1/2, which have been screened using qPCR assays (technological details are available upon request). The PAMs were prepared by our lab previously and stored in liquid nitrogen (49). PAMs were cultured with RPMI 1640 medium (Gibco, Carlsbad, CA, USA) containing 10% fetal bovine serum (Gibco) and maintained at 37°C in a humidified incubator with 5% CO_2_. The genotype II ASFV CN/GS/2018 strain, which was isolated and stored in our laboratory previously, was used in the animal challenge experiments in this study (50). The virus was propagated in porcine BMDMs. BMDMs were cultured with RPMI 1640 medium (Gibco) containing 10% fetal bovine serum (Gibco) and maintained at 37°C in a humidified incubator with 5% CO_2_. At 96 hpi, viral supernatant was collected after centrifugation at 3,000 rpm, at 4°C for 10 min, and filtered through a 0.22 µm pore size filter and stored in aliquots at −80°C. The viral titers were determined by performing the hemadsorption (HAD) assay, and the 50% HAD dose was calculated by using the method of Reed and Muench, as described previously (51), the HEK293T cells were obtained from the American Type Culture Collection (CRL-11268).

### Antibodies and inhibitors

The commercial antibodies used in this study included GRP78/BIP Rabbit Polyclonal Antibody (11587-1-AP, 1:1,000; Proteintech), Rabbit Anti-Phospho-PERK (Thr980) antibody (bs-3330R, 1:1,000; Bioss), PERK Rabbit pAb (A18196, 1:1,000; ABclonal), XBP1S-specific Polyclonal antibody (24868-1-AP, 1:1,000; Proteintech), Rabbit Monoclonal (EPR5253) to IRE1 (phosphoS724) (AB124945, 1:1,000; abcam), IRE1; ERN1 Polyclonal antibody (27528-1-AP, 1:1,000; Proteintech), ATF6 Polyclonal antibody (24169-1-AP, 1:1,000; Proteintech), Monoclonal Anti-Flag M2 antibody produced in mouse (F1804, 1:1,000; Sigma-Aldrich), Myc-Tag Rabbit mAb (C-terminal) (AE070, 1:1,000; ABclonal), MYC tag mouse monoclonal antibody (60003-2-1g, 1:1,000; Proteintech), Goat Anti-rabbit IgG (H + L) Cross-Adsorbed Secondary Antibody, AlexaFluor 488 (A-11008, 1:1,000; Thermo Fisher Scientific), Goat Anti-rabbit IgG (H + L) Highly Cross-Adsorbed Secondary Antibody, Alexa Fluor 594 (A11037, 1:1,000; Thermo Fisher Scientific), Goat Anti-mouse IgG (H + L) Cross-Adsorbed Secondary Antibody, Alexa Fluor 594 (A11005,1:1000, Thermo Fisher Scientific), Goat Anti-Mouse IgG (H + L) Superclonal Secondary Antibody, Alexa Fluor 488 (A28175, 1:1,000; Thermo Fisher Scientific), GAPDH mouse monoclonal antibody (TA802519M, 1:1,000; Origene), β-tubulin mouse monoclonal antibody (66240-1-1g, 1:1,000; Proteintech), and anti-β-actin monoclonal antibody (sc-8432, 1:1,000; Santa Cruz Biotechnology).

Ceapin-A7 (HY-108434), 4μ8C (HY-19707), and GSK2606414 (HY-18072) were obtained from Med Chem Express (MCE, USA). All of the inhibitors were dissolved in DMSO. PAM cells were pretreated with the inhibitors for 24 h and infected with ASFV at a multiplicity of infection (MOI) of 0.1 for 36 h.

### Infection procedures

The animals were inoculated intramuscularly with the indicated amounts of ASFV CN/GS/2018 or equal amounts of solvent control. The animals were slaughtered and dissected on 0 (uninfected solvent control), 1, 3, 5, or 7 days after infection with ASFV. All the challenged animals were monitored daily for clinical signs, and the rectal temperatures were measured daily until the death of the animals. At necropsy, multiple samples of 13 tissues, including the heart, liver, lung, spleen, kidney, stomach, small intestine (jejunum), large intestine (colon), bladder, mesenteric lymph nodes (MLNs), SLNs, inguinal lymph nodes (ILNs), and hepatogastric lymph nodes (HGLNs) of the dissected animals were collected at each time point. The obtained samples were used for the virology and histopathology analysis. The spleens, kidneys, and submandibular lymph nodes collected from the animals at 0, 3, 5, and 7 days after infection were used for preparing the protein profiling and subjected to proteomics analysis.

### Hematoxylin and eosin staining

Pigs were humanely sacrificed at different points after challenge, following which postmortem examinations were carried out. Tissue samples, including the heart, liver, lung, spleen, kidney, stomach, small intestine (jejunum), large intestine (colon), bladder, MLNS, SLNs, ILNs, and HGLNs, were collected. These samples were then fixed in 4% paraformaldehyde for 24 h, dehydrated, and paraffin-embedded. The treated tissues were sectioned into 4 micron slices and stained with hematoxylin and eosin for microscopic examination. The histological changes in each tissue section were recorded, and the severity of the lesions was graded on a scale of 0–4. This grading system took into account five rating areas: inflammation, necrosis, congestion, fibrosis, and other histological changes. The maximum score possible was 20 points. The histological examination of the tissues provided valuable insights into the effects of the treatment on the pigs’ organs and lymph nodes. The grading system allowed for a quantitative assessment of the lesions’ severity, providing valuable data for further analysis and comparison.

### LC-MS/MS for protein identification and quantitation

The resulting MS/MS raw files were processed using the MaxQuant search engine (v.1.6.5.0) against the Sus scrofa database (Sscrofa11.1) concatenated with the reverse decoy database. Trypsin/P was specified as a cleavage enzyme allowing up to two missing cleavages. Carbamidomethyl (C) was specified as a fixed modification. FDR was adjusted to <1%, and the minimum score for modified peptides was set to >40. The minimum peptide length was set at 7. For the quantification method, the match between runs was enabled. All the other parameters in MaxQuant were set to default values. A reverse decoy database was used to compute the false-positive rate caused by random matching, and a common contamination database was added to eliminate the influence of contaminated proteins in the identification results.

Student’s *t*-test was used to find out the differentially expressed proteins, and the significant *P* values were calculated. Fold change (FC) was calculated by dividing the mean values of the virus-infected group by that of the control group (FC = mean of virus-infected group / mean of the control group). Proteins with *P* < 0.05 and FC >1.2 were considered upregulated, and those with *P* < 0.05 and FC <0.83 were downregulated.

### Protein-protein interaction network and protein functional enrichment analysis

Gene Ontology enrichment and Kyoto Encyclopedia of Gene and Genomes (KEGG) pathway analyses for each interacted module were performed using DAVID (52, 53). STRING database (https://cn.string-db.org/) was used to construct the PPI network for DEPs. Interactions with confidence scores over 0.7 were used to construct the PPI network.

### GSVA

The GSEABase (v.1.44.0) package was selected to load the gene set file, which was downloaded and processed from the KEGG database (https://www.kegg.jp/) for performing GSVA. The signaling pathway activity score was further estimated and assigned to each sample by the GSVA (v.1.30.0) package (54). Differential pathway analysis was then performed by the limma (v.3.38.3) package (55).

### Methods to evaluate tissue and infection specificity

Stage and infection specificity were evaluated using the SPM. In this study, SPM is defined as

Each expression profile across 4 dpi and three tissues is transformed into a vector *X*:


$$X=(x_{K0},\;x_{k3,\;}x_{K5},\;x_{k7,\;}x_{L0},\;x_{L3,}\;x_{L5},\;x_{L7},\;x_{S0},\;x_{S3,\;}x_{S5},\;x_{S7}).$$

In addition, the expression specificity at a certain stage and tissue is transformed into another vector, $X_{exp}$:


$$X_{exp}=(0,\;0,\;0,\;0,\;0,\;0,\;0,\;0,\;0,\;0,\;0,\;x_{\text{stage\textbackslash{}\_tissue}}),$$

which stands for the expression profile at the time points in a certain tissue to be evaluated.

SPM is calculated as the cosine similarity between vectors *X* and $X_{exp}$:


$$SPM=\frac{X\;\cdot \;X_{exp}}{|X|\;\cdot |\;X_{exp}|},$$

where |*X*| and |*X*_exp_| stand for the model of each vector, respectively. SPM values range from 0 to 1, with higher values indicating more specificity at these time points in a certain tissue and vice versa. An SPM over 0.7 can be considered relatively high, indicating relatively specific expression at a certain stage in a specific tissue (56).

### RNA interference and qPCR analysis

The siRNA-control, si-Eif2AK3, si-ERN1, and si-ATF6 were synthesized from Tsingke (Beijing, China). Knockdown of genes in PAMs was performed using Namipo transfection reagent (TSBBR003; TranSheepBio, Shanghai, China). PAM cells (1 × 10^5^) were transfected with si-control, si-Eif2AK3, si-ERN1, and si-ATF6 (50 pM). Forty-eight hours after transfection, cells were left infected with ASFV at an MOI of 0.1 for 36 h.

Total RNA was isolated from whole tissue or PAM cells extracts, reverse transcribed, and quantified by RT-qPCR using the following primers listed in Table S1. The mRNA was extracted using RNA Isolator Total RNA Extraction (Vazyme), and cDNA was synthesized using HiScript II Q RT SuperMix for qPCR (Vazyme). qPCR was performed using the ChamQ Universal SYBR qPCR Master Mix (Vazyme) on the ABI StepOnePlus system. Expression was quantified using the 2−ΔΔCT method, and porcine glyceraldehyde 3-phosphate dehydrogenase (GAPDH) was used to normalize the expression of the target genes between samples.

### Plasmid construction and immunoprecipitation

The plasmid pHAGE-3*Myc-GRP78 was constructed by amplifying the full-length sequence of the porcine GRP78 gene using it as a template, and then cloning it into the phage vector pHAGE-BSD-3*Myc (NdeI-ApaI). The plasmid pHAGE-3*Flag-D117L was generated by cloning the ASFV D117L gene into the pHAGE-3*Flag expression vector, resulting in pHAGE-D117L-3*Flag with a 3*Flag tag at the C-terminal. The plasmid was constructed using a one-step cloning kit (Vazyme, Nanjing, China). The three mutants of pHAGE-3*Flag-D117L were obtained by using the following primers to perform deletion mutations based on it. Primers messages:

D117LΔ38-60-F: CGGAACAAACCGGACTATTGACTGCAAGTCGAG,

D117LΔ38-60-R: ATAGTCCGGTTTGTCCGGGATATTTGGCGATT,

D117LΔ67-76-F: CGGACTATTGACTGCTACTATGTACAACAACCTGAGCCTCA,

D117LΔ67-76-R: GTAGCAGTCAATAGTCCGGTTATAGTAAACGAT,

D117LΔ96-117-F: AAAAGGCATATGCGTCCGGCGTGCAAAATCCCG,

D117LΔ96-117-R: CGGACGCATATGCCTTTTTCTAAAGAATACCGGGAA.

DNA sequencing confirmed the expected specific sequence in the construct (Qingke, Xi'an, China).

For immunoprecipitation analysis, the HEK293T cells were homogenized with IP lysis buffer (containing 50 mM Tris-Cl [pH = 7.4], 150 mM NaCl, 1%Triton-100, 0.1% SDS, 1% sodium deoxycholate, and protease inhibitor cocktail), centrifuged to remove cell debris, and then the supernatant was collected for immunoprecipitation with Myc (HY-K0206, MCE) and DYKDDDDK magnetic beads (b26103; Selleck Chemicals, USA) at 4°C for 2 h. After washing five times with IP lysis buffer, the protein-bound beads were finally resuspended in SDS-PAGE loading buffer. The samples were boiled at 95°C for 10 min, and the supernatant was loaded on the gel for immunoblotting.

### Plasmid transfection and luciferase reporter assay

The following promoter reporter constructs are used to evaluate the ATF6 activation: pGL4.39(luc2P/ATF6 RE/Hygro) (E3661; Promega, USA), and the herpes simplex virus thymidine kinase promoter-Renilla luciferase reporter plasmid (pRL-TK). HEK293T cells were transfected with 10 ng pRL-TK, 50 ng ATF6, and 200 ng ASFV D117L using PolyPlus (101000046; Polyplus Transfection, France). At 24 h post-transfection, cells were washed with PBS and then lysed in luciferase lysis buffer. The reporter activity was measured using the Dual-luciferase Reporter Assay System (E1960, Promega).

### Immunofluorescence

IPAM cells were fixed in 4% formaldehyde for 10 min, permeabilized with 0.5% Triton X-100 for 10 min, and then blocked in 5% bovine serum albumin in PBS for 2 h at room temperature. Primary antibodies against MYC, FLAG, and HSPA5 were added to the PBS and incubated at 4°C overnight. Subsequently, the samples were washed with PBST (0.1% Tween 20) and incubated with the appropriate fluorescently labeled secondary antibodies for 2 h at room temperature. Following three washes with PBST, DAPI (D9542-5mg; Sigma-Aldrich, USA) was used to stain the nucleus for 2 min. Then, washing with PBST three times, a Zeiss confocal microscope (LSM980) was used to take photos.

### Statistical analysis

Data are presented as means ± standard error. Statistical analysis was evaluated using Student’s *t*-tests in R (v.4.1.2) as indicated in the figure legends. Statistical significance was defined as *P < 0.05, **P < 0.01, and ***P < 0.001 versus controls.

## Data Availability

The mass spectrometry proteomics data have been deposited at the ProteomeXchange Consortium via the PRIDE (57) partner repository with the data set identifier PXD047161.

## SUPPLEMENTAL MATERIAL

Supplemental material mbio.03242-25-s0001.docx Fig. S1 to S3; Table S1.

ASM does not own the copyrights to Supplemental Material that may be linked to, or accessed through, an article. The authors have granted ASM a non-exclusive, world-wide license to publish the Supplemental Material files. Please contact the corresponding author directly for reuse.
