# Supplementary material for: Quantitative proteomic analysis identifies the unfolded protein response as a host pathway co-opted by ASFV to promote replication
Source: mBio. 2025 Dec 5;17(1):e03242-25. doi: 10.1128/mbio.03242-25 (PMC12802178; doi:10.1128/mbio.03242-25)
Supplement: Supplemental material — Fig. S1 to S3; Table S1. [file mbio.03242-25-s0001.docx]

**Quantitative proteomic analysis identifies the unfolded protein response as a host pathway co-opted by ASFV to promote replication**

Danyang Zhang^1,#^, Baohong Liu^1,#^, Huanan Liu^1,2^, Ruoqing Mao^1,2^, Weijun Cao^1,2^, Xiangle Zhang^1^, Fayu Yang^1,3^, Yichao Wang^1,3^, Chaochao Shen^1,2^, Shilei Zhang^1,3,^*, Zixiang Zhu^1,2,3,4,^*, and Haixue Zheng^1,2,3,4,^*

1. State Key Laboratory for Animal Disease Control and Prevention, College of Veterinary Medicine, Lanzhou University, Lanzhou Veterinary Research Institute, Chinese Academy of Agricultural Sciences, Lanzhou 730046, China
2. African Swine Fever Regional Laboratory of China, Lanzhou Veterinary Research Institute, Chinese Academy of Agricultural Sciences, Lanzhou 730046, China
3. Key Laboratory of Animal Virology of the Ministry of Agriculture, Lanzhou Veterinary Research Institute, Chinese Academy of Agricultural Sciences, Lanzhou 730046, China

4. Gansu Province Research Center for Basic Disciplines of Pathogen Biology,

Lanzhou 730046, China.

*Corresponding author:

Shilei Zhang, ZhangSL8600@outlook.com

Zixiang Zhu, zhuzixiang@caas.cn

Haixue Zheng, zhenghaixue@caas.cn

#Co-first author

**Supplementary figures**


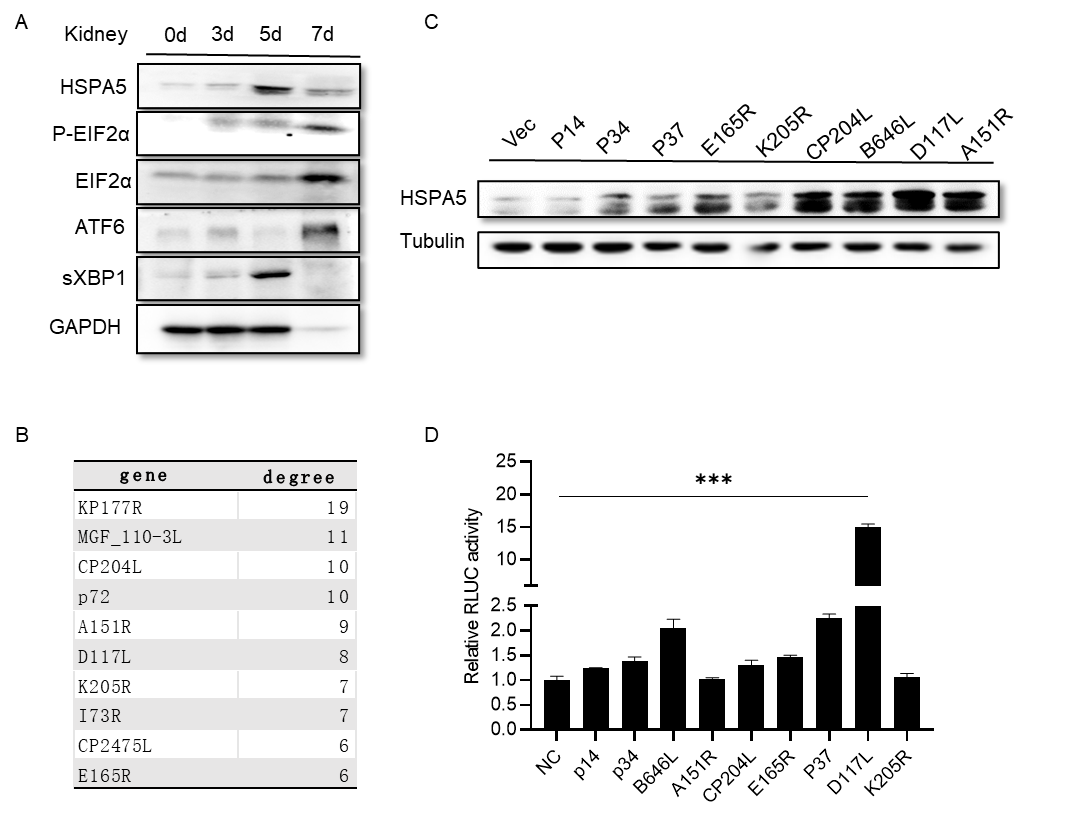


**Supplementary Figure 1. Screening of ASFV proteins induced UPR response**

**A.** UPR signaling pathway activation in the Kidney upon ASFV infection was examined by immunoblotting assay.

**B.** Top 10 ASFV proteins interact with host UPR related proteins based on PPI network analysis.

**C.** HEK293T cells were pre-treated with DMEM medium containing 2% FBS in a starvation condition for 12 hours, then transfected above-mentioned ASFV plasmids, HSPA5 protein expression was detected by Western blot at 24h. Data are presented as mean + SD. P values were calculated by a two-tailed unpaired t-test. *P < 0.05, **P < 0.01, ***P < 0.001.

**D.** HEK293T cells were transfected with vector, Flag-p14, Flag-p34, Flag-B646L, Flag-A151R, Flag-CP204L, Flag-E165R, Flag-p37, Flag-D117L or Flag-K205R, each group was co-transfected with ATF6-Luc and Renilla-TK. Detection of luciferase activity of ATF6 by Dual-luciferase assay.


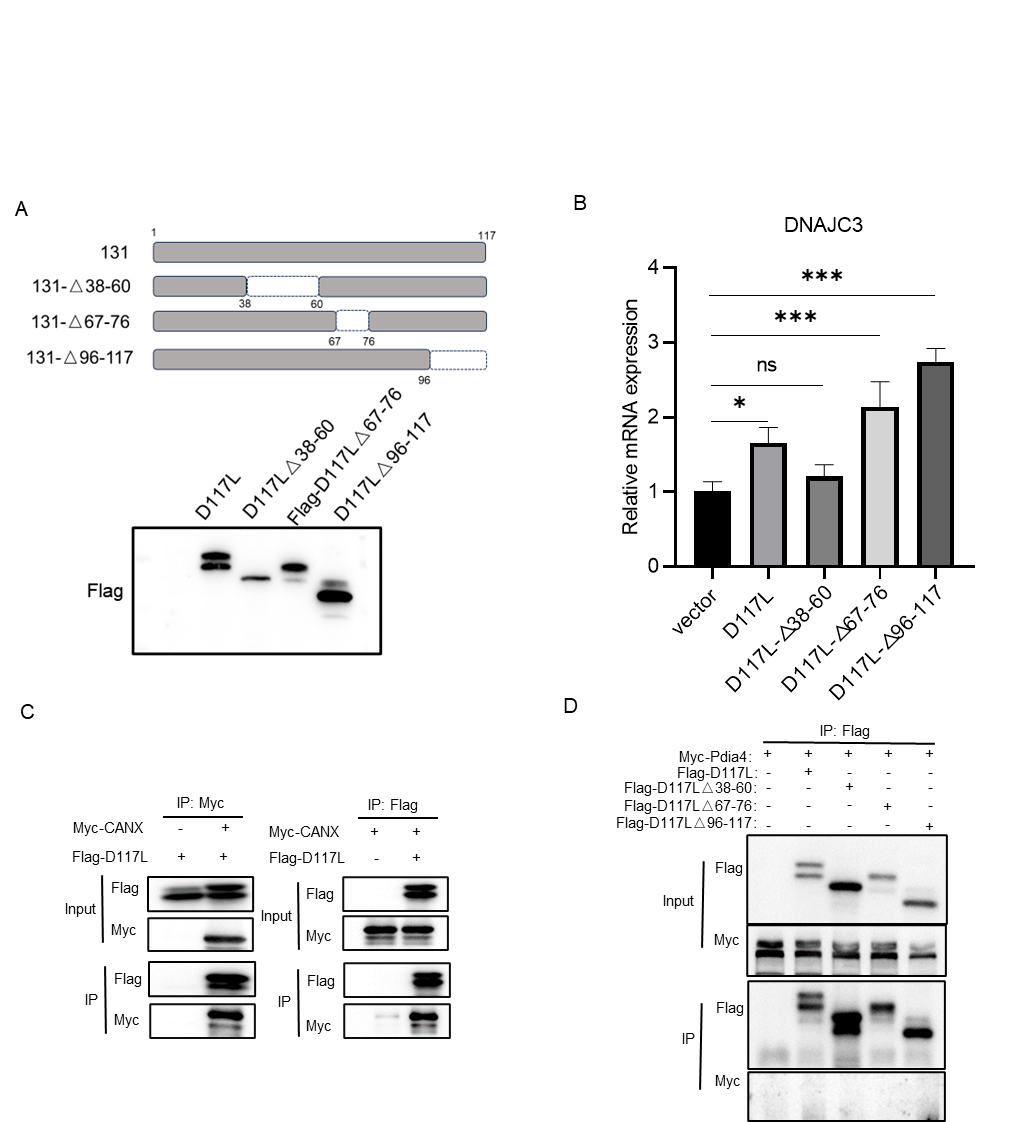


**Supplementary Figure 2. Identification of the key domain of D117L in activating UPR**

**A.** The ASFV D117L protein consists of 117 amino acids. Based on its sequence, Flag-D117L△38-60, Flag-D117L△67-76 and Flag-D117L△96-117 were constructed. HEK293T cells were transfected D117L (WT) or mutants to verify their expression.

**B.** HEK293T cells were transfected with vector, Flag-D117L or different mutants for 24h, mRNA expression of Dnajc3 was detected by RT-qPCR.

**C.** Co-IP assays assessed the interaction of CANX with D117L

**D.** Co-IP assays assessed the interaction of PDIA4 with D117L (WT) or mutants in HEK293T cells.


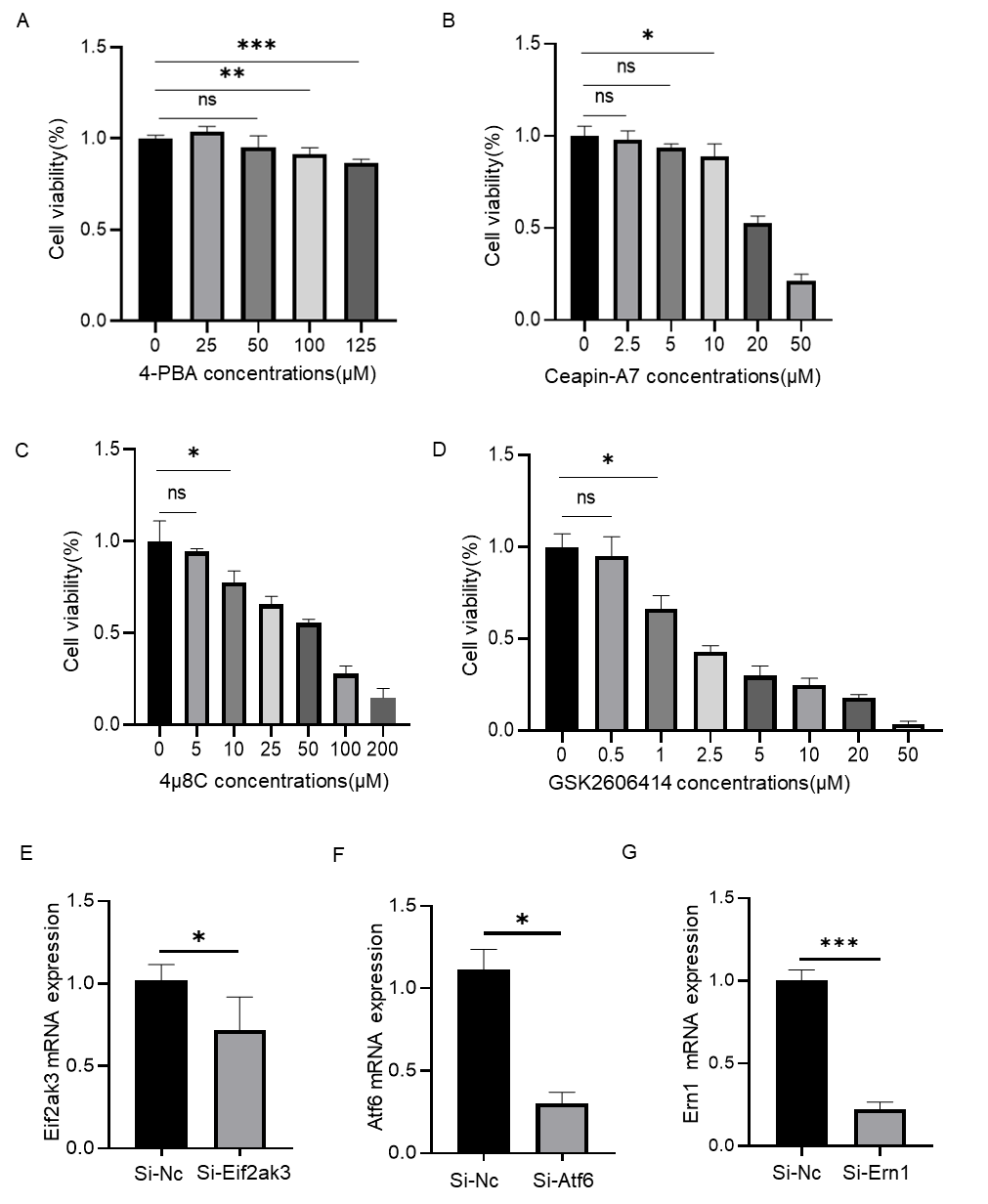


**Supplementary Figure 3. Screening of the optimal treatment concentrations of the inhibitors 4-PBA, Ceapin-A7, 4μ8C, and GSK2606414 and evaluation of the efficacy of small interfering RNAs (siRNAs).**

**A-D.** PAM cells were treated with 4-PBA (0, 25,50,100 or 125 μM), Ceapin-A7 (0, 2.5, 5, 10, 20, 50μM), 4μ8C (0,5,10,25,50,100,200μM) or GSK2606414 (0, 0.5, 1, 2.5, 5, 10, 20, 50μM) for 24h. Cell viability was determined by CCK-8 analysis.

**E-F.** The RNA interference efficacy was detected by RT-qPCR. Data are presented as mean+SD. P values were calculated by a two-tailed unpaired t-test. *P < 0.05, **P < 0.01, ***P < 0.001.

**Supplementary Table 1：Primers used in this study**

| **Primers** | **Sequences (5′-3′)** |
| --- | --- |
| ASFV B646L- F | CCGGGTACAATGGGTCTTCC |
| ASFV B646L- R | CGCAACGGATATGACTGGGA |
| ASFV CP204L-F | CTCCGATGAGGGCTCTTGCT |
| ASFV CP204L-R | AGACGGAATCCTCAGCATCTTC |
| Sus scrofa-HSPA5-F | ACTCGTGGTAAGTGGGGTTG |
| Sus scrofa-HSPA5-R | TTCTTGAACACCCCAACGCT |
| Sus scrofa-ATF6-F | CTCGACAGCGTTTCGGAGTA |
| Sus scrofa-ATF6-R | GCCAAGTTCAGCAAACAGGG |
| Sus scrofa-DDIT3-F | GCTCTGATTGACCGGATGGT |
| Sus scrofa-DDIT3-R | GGTTGGTAGCCACTTCCAGG |
| Sus scrofa-EIF2AK3-F | GTGGGATTTGGACGTGGGAT |
| Sus scrofa-EIF2AK3-R | AGCTCTCACGGTTTTCTGGG |
| Sus scrofa-ATF4-F | CTTGATGTCCCCCTTCGACC |
| Sus scrofa-ATF4-R | TGTCTGAGGCACTGACCAAC |
| Sus scrofa-ERN1-F | CTGCACTCCCTCAACATCGT |
| Sus scrofa-ERN1-R | GTAGGTGGGGTTCTCCTTGC |
| Sus scrofa-GAPDH-F | CCACTGGTGTCTTCACGACC |
| Sus scrofa-GAPDH-R | GGTTCACGCCCATCACAAAC |
| Sus scrofa-DNAJC3-F | GAAAAGGAAGCCCAGTCCCA |
| Sus scrofa-DNAJC3-R | CATTGTCAGGCTCCACCTGT |
| Sus scrofa-DNAJB9-F | GTGGGCCAGAACCAATAGGG |
| Sus scrofa-DNAJB9-R | CATCGAACAACCCACCTCCA |
